# Epinephrine in Neonatal Resuscitation

**DOI:** 10.3390/children6040051

**Published:** 2019-04-02

**Authors:** Payam Vali, Deepika Sankaran, Munmun Rawat, Sara Berkelhamer, Satyan Lakshminrusimha

**Affiliations:** 1 UC Davis School of Medicine, Sacramento, CA 95817, USA; slakshmi@ucdavis.edu; 2 SUNY Buffalo, Buffalo, NY 14222, USA; deepikasnkrn@gmail.com (D.S.); mrawat@upa.chob.edu (M.R.); saraberk@buffalo.edu (S.B.)

**Keywords:** resuscitation, epinephrine, newborn, intravenous, intraosseous, intramuscular

## Abstract

Epinephrine is the only medication recommended by the International Liaison Committee on Resuscitation for use in newborn resuscitation. Strong evidence from large clinical trials is lacking owing to the infrequent use of epinephrine during neonatal resuscitation. Current recommendations are weak as they are extrapolated from animal models or pediatric and adult studies that do not adequately depict the transitioning circulation and fluid-filled lungs of the newborn in the delivery room. Many gaps in knowledge including the optimal dosing, best route and timing of epinephrine administration warrant further studies. Experiments on a well-established ovine model of perinatal asphyxial cardiac arrest closely mimicking the newborn infant provide important information that can guide future clinical trials.

## 1. Introduction

The infrequent need for chest compressions and epinephrine use during neonatal resuscitation [1,2], coupled with an inability to consistently anticipate which newborns are at high risk of requiring extensive resuscitation, explains the ongoing lack of high quality evidence supported by large randomized clinical trials to better guide healthcare providers in their resuscitative efforts. The current understanding and knowledge of resuscitative medicine in newborns is further limited by animal and simulation models that do not adequately depict the transitioning fetal circulation, fluid-filled alveoli and patent ductus arteriosus inherent to newborn infants [3,4,5]. Furthermore, the underlying etiology of bradycardia, and ultimately cardiac arrest, in neonates, as a result of severe hypoxemia, metabolic acidosis and vascular compromise, contrasts that which is most commonly observed in adults, where the abrupt cessation of cardiac output in the setting of well-oxygenated blood follows the onset of arrhythmias. The current recommendations guiding neonatal resuscitation with regard to chest compressions and epinephrine administration are largely extrapolated from studies compromised by the aforementioned limitations. Additional studies evaluating chest compressions and epinephrine in a model with transitioning physiology and fluid-filled lungs may potentially impact the use of these interventions [6].

Severely asphyxiated neonates with extreme bradycardia or cardiac arrest who have been successfully resuscitated following chest compressions and (or) epinephrine administration are at greater risk of severe neurologic impairment [7,8,9]. While the ultimate goal of resuscitative efforts is to swiftly establish the return of spontaneous circulation (ROSC), assuring adequate perfusion to vital organs by means of efficient chest compressions and vasoactive drug administration are likely to improve intact survival. Epinephrine is the only medication currently recommended for neonatal resuscitation by the International Liaison Committee on Resuscitation (ILCOR) [10,11,12]. The optimal timing, route and dose of epinephrine administration in neonatal resuscitation, however, has not been established. In the following review, a brief summary on the evidence of epinephrine use in neonatal resuscitation, as well as data from emerging translational studies from an ovine model of transitioning physiology and fluid-filled lungs is presented. We also reviewed the current understanding of dose, route and efficacy of epinephrine and alternate medications that have been investigated for neonatal bradycardia and cardiac arrest.

## 2. A Brief History on Coronary Perfusion Pressure and Epinephrine

In the late 19^th^ century, physiologic research showed that the excised heart could beat again when the coronary arteries were subjected to a considerable pressure from some circulating medium [13]. During the same period, scientists studying the physiologic effects of an isolated substance (later termed epinephrine also known as adrenaline [14]) from the suprarenal capsules (adrenal glands) demonstrated significant increases in heart rate and blood-pressure following its intravenous (IV) administration in experimental dogs [15]. Following the discovery of epinephrine’s pharmacologic effects and recognizing the importance coronary pressures play in reviving the heart, the value of administering epinephrine to raise coronary pressures was soon appreciated. The use of epinephrine did not become clinically widespread until the 1960s, however, breakthrough experiments by Redding and colleagues demonstrated a significant improvement in rates of ROSC with the administration of IV epinephrine in a canine model of asphyxia-induced cardiac arrest [16].

**Figure 1:**
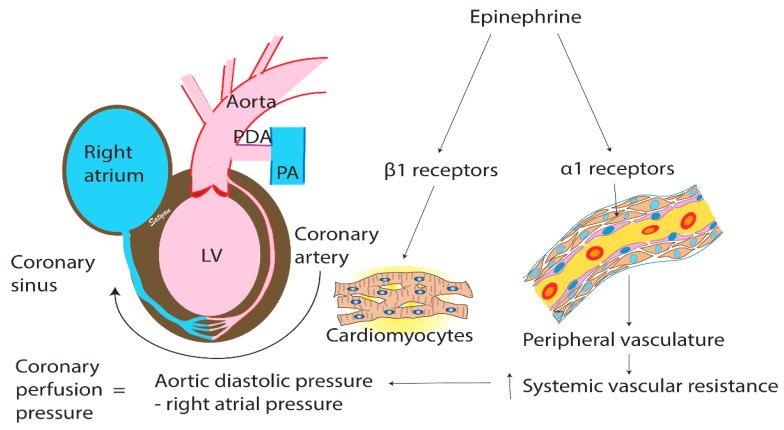
Coronary perfusion pressure (CPP) and the mechanism of action of epinephrine. CPP is calculated as the difference between the aortic diastolic pressure and the right atrial pressure serves as a surrogate to coronary blood flow. In the premature infant, the effect of a left to right (from aorta into pulmonary artery) flow on coronary blood flow is unknown. Epinephrine’s effect on alpha-adrenergic receptors on peripheral vasculature leads to vessel contraction and a rise in systemic vascular resistance that can increase CPP. Epinephrine also exerts stimulation of beta-adrenergic receptors on myocytes that increase cardiac contractility. α: Alpha; β: Beta; LV: Left ventricle; PA: Pulmonary artery; PDA: Patent ductus arteriosus. Copyright Satyan Lakshminruismha.

Catecholamines mediate their cardiovascular actions predominantly through α_1_, β_1_, β_2_, and dopaminergic receptors, the density and proportion of which modulate the physiological responses in individual tissues [17]. Epinephrine is an endogenous catecholamine with high affinity for α_1_, β_1_, and β_2_-receptors present in cardiac and vascular smooth muscle (Figure 1). Experimental studies in asphyxiated dogs pretreated with α-adrenergic inhibition (phenoxybenzamine) or β-adrenergic inhibition (propranolol) have demonstrated that α-adrenergic stimulation by epinephrine likely explains its mechanism of action [18], and epinephrine’s vasoconstrictive properties are primarily responsible for its effectiveness in achieving ROSC [19].

## 3. Epinephrine in Neonatal Resuscitation

In the asphyxiated, severely acidotic state, the newborn is likely to be maximally vasodilated with very low systemic vascular resistance (SVR). Administration of epinephrine is believed to induce intense peripheral vasoconstriction resulting in elevated SVR and an increase in coronary perfusion pressure (CPP) to improve coronary blood flow [2,20]. However, in severely acidotic lambs (by infusion of lactic acidosis), hemodynamically compromised through hypoxemia, intravenous epinephrine administration at 0.01 mg/kg did not improve cardiac output, heart rate or blood pressures [21]. Not only is the efficacy of epinephrine use in neonatal resuscitation poorly understood, the optimal timing, dose and route, and the potential adverse effects of epinephrine administration remain largely unknown.

**Figure 2:**
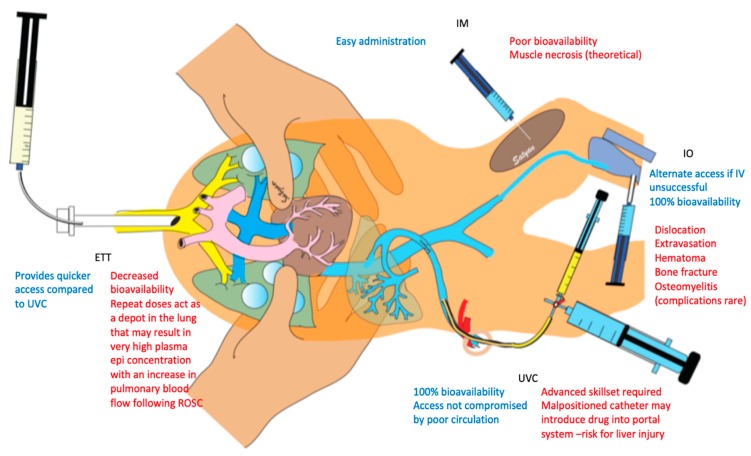
Infographic showing alternative routes of epinephrine administrations. Advantages are shown in blue and disadvantages in red. ETT: Endotracheal tube; IM: Intramuscular; IO: Intraosseous; IV: Intravenous; UVC: Umbilical venous catheter. Copyright Satyan Lakshminrusimha.

Intravenous (IV) administration of epinephrine is preferred as it provides 100% bioavailability. Alternate routes of drug administration have been described as early as 1913 through an endotracheal tube (ETT) [22] or an intraosseous (IO) device, first reported in the literature in 1916 [23], and by intramuscular (IM) injections (a common route during anaphylaxis) (Figure 2). The current recommended epinephrine dose by the neonatal resuscitation program (NRP) is 0.01–0.03 mg/kg IV or IO and 0.05–0.1 mg/kg through the ETT [24]. 

### 3.1. Intravenous Epinephrine

In their canine model of asphyxial cardiac-arrest model, the authors reported greater success of ROSC compared to normal saline following administration of epinephrine at 1 mg [16]. Clinical studies following this report did not account for the weight difference and showed the return of spontaneous circulation at the same dose (i.e., ≈ 0.01–0.015 mg/kg, assuming an adult weight of 70 kg), which was then extrapolated to neonatal and pediatric patients with dose ranges of 0.01–0.03 mg/kg. High-dose IV epinephrine (0.1–0.2 mg/kg) in newborn and pediatric animal models has been shown to be associated with severe tachycardia, hypertension, reduced stroke volume and cardiac output, and higher mortality in the immediate post-resuscitation period [25,26]. The strongest evidence regarding high- or low-dose epinephrine comes from a randomized clinical study of in-hospital pediatric cardiac arrest. Following a first standard-dose of epinephrine at 0.01 mg/kg IV, 68 patients were randomized to receive subsequent doses of either 0.1 mg/kg or 0.01 mg/kg. Success in ROSC was similar between groups (20/34 and 21/34, respectively), however there were no survivors in the patients randomized to high-dose epinephrine compared to 4/34 (12%) in the low-dose group [27]. Furthermore, among the 30 patients with asphyxial cardiac arrest, 7/18 patients randomized to low-dose epinephrine survived, whereas none of the 12 patients randomized to high-dose epinephrine survived to hospital discharge. The American Heart Association currently dissuades the use of high-dose IV epinephrine in adult cardiac arrest [28].

#### Low Umbilical Venous Catheter

**Figure 3:**
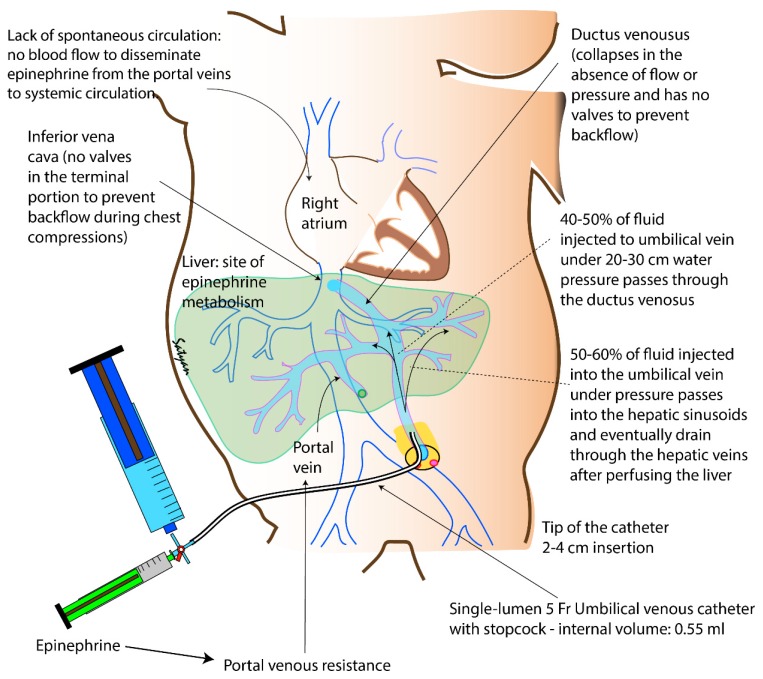
Causes for poor bioavailability and efficacy of epinephrine administered through a low umbilical venous catheter (UVC). The internal volume of a 5 Fr UVC with a stopcock is 0.55 mL. A flush of 0.5–1 mL will clear the catheter and deposit epinephrine in the umbilical vein. In the presence of adequate pressure and flow into the umbilical vein, 40–50% of administered fluid passes through the ductus venosus [30]. In the absence of umbilical flow (as in cardiac arrest), the inlet of the ductus venosus narrows. The terminal portion of the inferior vena cava and ductus venosus do not have valves and backpressure from chest compressions can potentially cause back-flow. Epinephrine also increases portal venous resistance. The liver is also a major site of epinephrine breakdown. Right atrial delivery of a vasopressor can be enhanced by (a) catheter placement in the right atrium (not feasible in the delivery room); (b) quick flush with a mini-bolus to open up the ductus venosus and enhanced delivery to the heart in the absence of spontaneous circulation. Copyright Satyan Lakshminrusimha.

Placement of a low UVC (2–4 cm from the umbilical stump) is an effective and efficient mode of securing IV access in the delivery room. The bioavailability and plasma concentrations achieved with administration of epinephrine by this route are similar to that of epinephrine delivered via a central venous catheter. The umbilical vein joins the left branch of the portal vein and eventually drains through the ductus venosus in to the inferior vena cava. Animal experiments demonstrate that 50% of umbilical blood flow is shunted through the ductus venosus. During hypoxemia, the shunted fraction could reach 70%, especially when associated with hypovolemia. In human fetuses, the shunted fraction is 28–32% at 18–20 weeks gestation, 22% at 25 weeks and 18% at 31 weeks. Based on the concept of the “via sinistra” pathway, blood is preferentially streamed across the oval foramen to the left atrium, left ventricle, ascending aorta and coronary circuit [29]. It is likely that epinephrine administered through a UVC enters the left side of the heart through the patent foramen ovale subsequently accessing the systemic circulation (and coronary circulation). Thus, epinephrine administered by this route bypasses the liver and is not subject to hepatic metabolism (Figure 3).

Epinephrine administration by a low umbilical venous route has several advantages and is the preferred route as per NRP recommendations; these advantages include:

- Ease of placement by trained resuscitators
- 100% bioavailability
- Bypass of hepatic metabolism if drug enters inferior vena cava through the ductus venosus.
- Access to the systemic circulation through an oval foramen and bypass of the lung
- Efficacy in clinical and translational studies
- Benefit of venous access for the administration of volume bolus (including transfusion of packed red blood cells) and blood sampling.

There are some drawbacks of umbilical venous epinephrine; these include:

- Placement of the catheter requires training. Adult providers such as emergency medical technicians are often uncomfortable placing an umbilical venous catheter.
- Access to a sterile catheter and insertion equipment is necessary.
- Complications of deep placement in a branch of the portal vein can include hepatic ischemia and possibly necrosis.
- Placement can be challenging. The ductus venosus has some inherent resistance in the absence of blood flow (as in cardiac arrest) and this resistance needs to be overcome with adequate volume of flush following administration of epinephrine.
- Attempts to place a UVC can interfere with the delivery of effective chest compressions. This can be overcome by delivering chest compressions from the head-end of the radiant warmer.

### 3.2. Endotracheal Epinephrine

IV access is not always readily available. In a simulation study to assess timing of epinephrine dose, time to place an umbilical venous catheter (UVC) took a mean of 6 min compared to a mean intubation time of less than 2 min [31]. Therefore, while intravenous access is attempted, NRP recommends giving epinephrine through the ETT as an alternative route. In a retrospective study evaluating the efficacy of ETT epinephrine in the delivery room (at a time when the recommended dose was 0.01–0.03 mg/kg), 94% (44/47) of newborns requiring cardiopulmonary resuscitation (CPR) received the first dose via ETT with a ROSC success of only 32% (14/44) [1]. In a more recent retrospective study, only 20% (6/30) of newborns in the delivery room who received ETT epinephrine at a dose of 0.03 or 0.05 mg/kg achieved ROSC, while 71% (17/24) were subsequently successfully resuscitated following IV epinephrine administration [32,33,34]. 

The high frequency of initial ETT epinephrine use in clinical practice makes it imperative that the recommended dose be as effective as possible. In a newborn piglet model with induced ventricular fibrillation (VF), there was no significant increase in plasma epinephrine concentration as compared to normal saline controls following ETT epinephrine at a dose of 0.01 mg/kg [35]. Similarly, in an adult swine model of VF, administration of epinephrine by ETT at 0.01 mg/kg did not result in any meaningful rise in epinephrine plasma concentrations compared to normal saline control animals, whereas ETT epinephrine at 0.1 mg/kg resulted in concentrations of 215 ± 40 ng/mL with repeat ETT dosing doubling the concentration to 402 ± 80 ng/mL [36]. Interestingly, instillation of ETT epinephrine at 0.1 mg/kg in isolated rabbit lung models has shown a decreased effect on the pulmonary vascular response and lower epinephrine concentrations in lungs isolated from rabbits aged 1 to 3 days compared to those aged 14 to 21 days [37]. The presence of thicker vascular and alveolar walls in the newborn favors using a higher dose of ETT epinephrine. 

**Figure 4:**
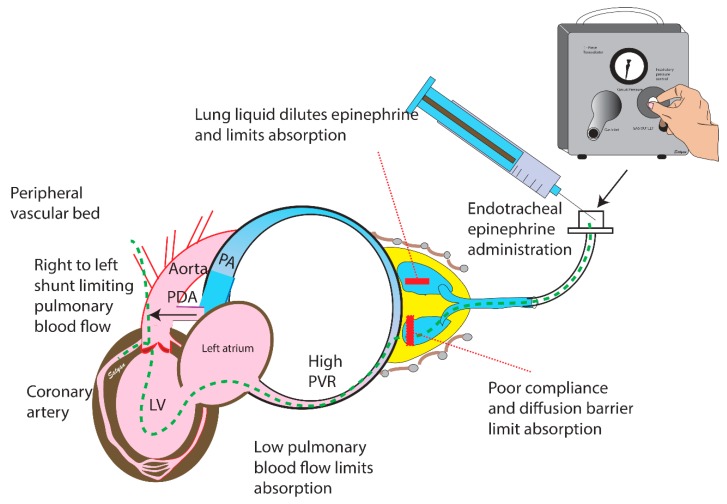
Endotracheal epinephrine. Asphyxia and acidosis decrease systemic vascular resistance by dilating the peripheral vascular bed, and high fetal pulmonary vascular resistance may lead to right to left shunting at the PDA limiting pulmonary blood flow. The presence of fetal lung liquid may dilute tracheal epinephrine, and absorption is further compromised by low pulmonary blood flow. The dashed green line represents the proposed path of intratracheal epinephrine. A higher dose of endotracheal epinephrine may compensate for dilution of lung liquid and overcome the diffusion barrier to achieve higher plasma concentrations. LV: Left ventricle; PA: Pulmonary artery; PDA: Patent ductus arteriosus; PVR: Pulmonary vascular resistance. Copyright Satyan Lakshminrusimha.

Recently, experiments in newborn lambs with transitioning fetal circulation and fluid-filled lungs that closely mimic hemodynamically compromised newborns in the delivery room have proven helpful in better understanding the effects and pharmacokinetics of epinephrine in neonatal resuscitation [38] (Figure 4). In this perinatal asphyxial cardiac arrest lamb model, ETT epinephrine administration at 0.1 mg/kg resulted in delayed and lower peak plasma epinephrine concentrations (130 ± 60 ng/mL at five minutes) compared to IV epinephrine administration at 0.03 mg/kg (≈ 460 ± 210 ng/mL at one minute). The epinephrine concentration following ETT epinephrine was also considerably lower than the values reported by the aforementioned study on adult swine [36]. This would be expected as several limitations, particularly in the newborn, may decrease epinephrine absorption from the lungs: (1) The fluid-filled lungs may dilute the drug, (2) high pulmonary vascular resistance and extracardiac shunts decrease pulmonary blood flow, (3) the epithelial linings of the respiratory bronchi, alveoli, and pulmonary capillaries are relatively thick at birth, and (4) epinephrine may cause local pulmonary vasoconstriction limiting its own absorption [39]. Success of ROSC was also lower in the ETT group (12/22 or 55%) compared to the intravenous group (19/22 or 86%). Seven lambs in the ETT group that did not initially achieve ROSC were successfully resuscitated following IV epinephrine. The limited absorption of ETT epinephrine also highlights the fundamental principle of newborn resuscitation: Ventilation and achieving adequate functional residual capacity (FRC). Pulmonary vascular resistance (PVR) is minimal at FRC [40]. Progressive increases in mean airway pressure towards total lung volume reduces cardiac output by decreasing venous return and compressing alveolar pulmonary vessels. Underinflated lungs may kink extra-alveolar pulmonary vessels and increase PVR. Achieving an optimal FRC during ventilation maximizes pulmonary blood flow and may enhance absorption of endotracheal epinephrine.

### 3.3. Intraosseous Epinephrine

**Figure 5:**
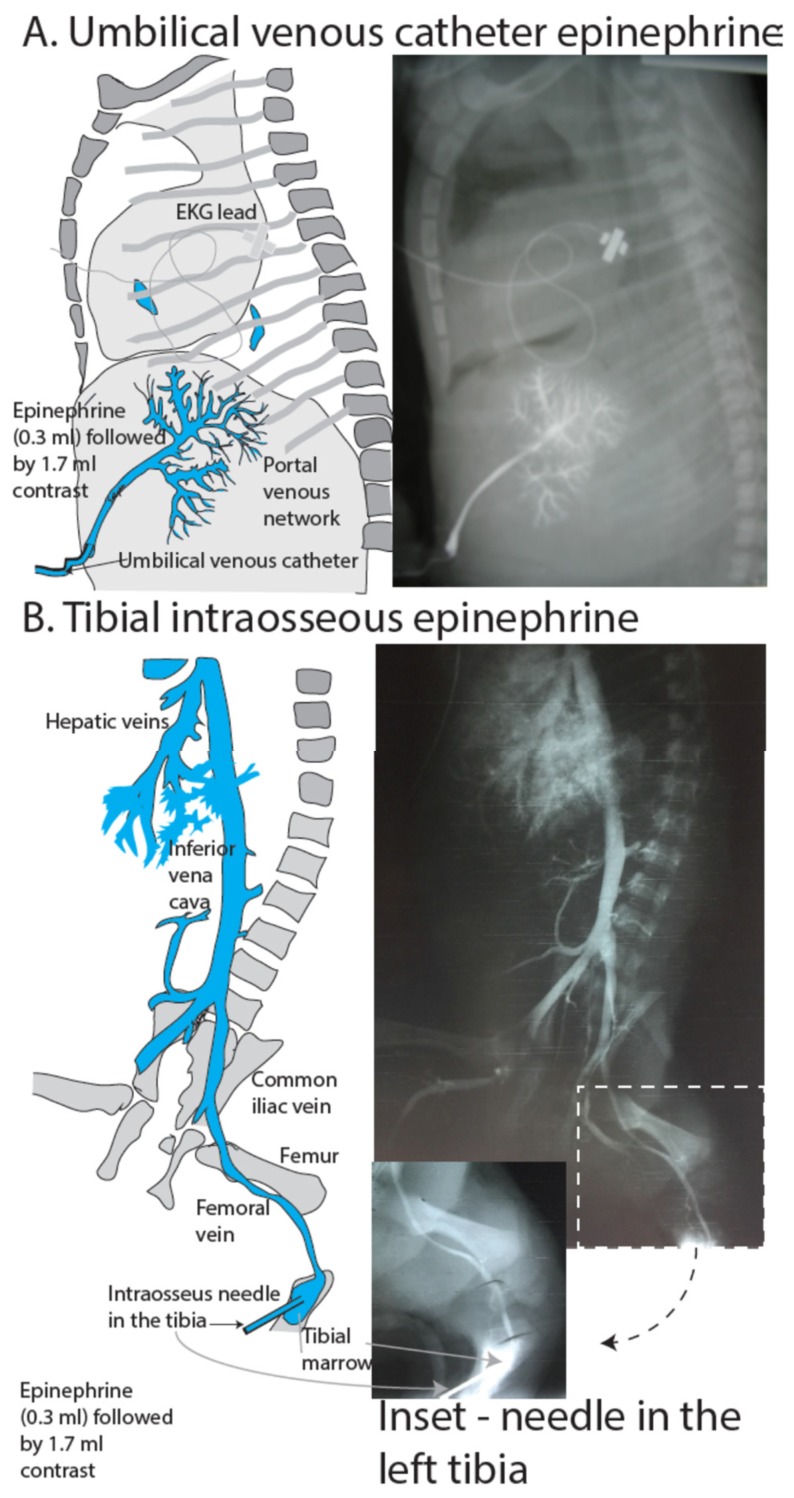
X-ray showing distribution following (**A**) low-lying umbilical venous catheter or (**B**) tibial IO administration of contrast in a perinatal cardiac arrested lamb model following 30 sec of chest compressions. Rapid distribution of contrast into the venous vasculature and heart can be appreciated following IO administration. IO: Intraossesus. Copyright Satyan Lakshminrusimha.

Neonatal and pediatric resuscitation guidelines recommend IO epinephrine administration in cases where IV access is unsuccessful [24,41]. In a neonatal simulation study, insertion of an IO device was quicker by a mean of 46 seconds compared to placement of a UVC [42]. The medullary space in the epiphyseal plate of long bones has a rich blood supply, which remains well perfused during shock and hypotension [43]. Adult and animal pharmacokinetic studies have shown equivalent pharmacokinetics comparing IV to IO drug administration [44,45]. Animal studies report contradicting results about pharmacokinetics and plasma availability of IO epinephrine administration [46,47]. In a porcine model of cardiac arrest, peak epinephrine concentrations were achieved quicker following IV administration (78 ± 69 sec) compared to IO administration (156 ± 13 sec) [47]. In a noncardiac arrest lamb model, a similar linear increase and comparable peak plasma epinephrine concentrations have been observed following IO and IV administration [46]. Several cases of IO epinephrine administration in neonates, including extreme premature infants have been reported in the literature with success in achieving ROSC [48,49,50,51]. There are several different IO devices available, including manual and semi-automatic types (e.g., Cook intraosseous needle [Cook Medical, Bloomington, IN, USA], EZ-IO [Telefex Medical, Toronto, Canada]) [52]. IO epinephrine, therefore, may be a superior alternative to ETT epinephrine when IV access is difficult or the skillset for UVC insertion is lacking. The efficacy of IO epinephrine in the context of transitional physiology and asphyxia, however, needs further study (Figure 5).

### 3.4. Intramuscular Epinephrine

Obtaining IV, IO and (or) ETT access can often be challenging and requires an advanced skillset by medical caregivers. IM injection, however, is a simple procedure that can be performed by a layperson. IM epinephrine is well established as the initial treatment of choice for systemic anaphylaxis [53]. The ease of IM epinephrine injections offers a potential alternate route of epinephrine administration for newborn resuscitation, particularly in less resourced environments where obtaining IV or IO access may be more difficult. The literature on the use of IM epinephrine for resuscitation is sparse. In a pilot study in a swine model of cardiac arrest, IM epinephrine (0.1 mg/kg) compared to IV epinephrine (0.01 mg/kg) demonstrated a comparable success in achieving ROSC [54]. However, no data on pharmacokinetics and plasma epinephrine concentrations were reported. There are currently no studies evaluating epinephrine absorption following IM administration in newborn resuscitation. In a lamb model of perinatal asphyxia cardiac arrest, pharmacokinetic data from two lambs that were given IM epinephrine (0.1 mg/kg) in the deltoid muscle revealed that there was no significant rise in plasma epinephrine concentration. In a state of complete circulatory arrest and severe acidosis, chest compressions may not provide adequate perfusion to the muscles to circulate epinephrine deposited in the muscle. Future studies assessing the efficacy of IM epinephrine in a model of profound bradycardia (as opposed to asystole) are warranted. 

### 3.5. Efficacy of Epinephrine and Adverse Effects

The evidence on the efficacy of epinephrine in neonatal resuscitation is inconsistent owing to the heterogeneity of experimental models that differ in the species studied (dogs, piglets and lambs), the definition used for cardiac arrest (asystole or predefined hypotension/bradycardia), and the timing of epinephrine administration. The effects of epinephrine and chest compressions in asphyxia models [55,56,57,58,59,60,61,62,63,64,65] characterized by profound acidosis and hypoperfusion cannot easily be compared to VF models. Furthermore, animal asphyxia models wherein resuscitation is initiated following cardiac arrest (asystole) [60,61,62,63,64,65], as opposed to after a predetermined drop in heart rate or blood pressure [55,56,57,58,59,66], would be expected to show different results as bradycardic/hypotensive subjects are less acidotic, and may have a less compromised vascular tone. These limitations make the interpretation of data challenging. Epinephrine administration is not without risk; knowing when and if epinephrine may be beneficial with newborn resuscitation remains to be determined.

Focusing on studies in which animals were asphyxiated to cardiac arrest, Berg et al. have shown that 7/10 piglets in the chest compression and ventilation group achieved ROSC by the end of an 8-minute period of bystander CPR prior to any epinephrine administration [67]. In a study by McNamara et al. comparing a single dose of vasopressin (high or low dose –HDV, LDV) and epinephrine (high [0.03 mg/kg] or low [0.01 mg/kg] dose –HDE, LDE) to saline (control), 9/65 (14%) piglets achieved ROSC with the initiation of CPR prior to the administration of the study drug. In the remaining 56 piglets analyzed, the success of ROSC was similar in the control (5/12 or 42%) and the LDE group (5/13 or 39%), while HDE achieved ROSC in 6/11 (55%) piglets [56]. In perinatal asphyxial cardiac arrest lamb models comparing 3:1 compression-to-ventilation CPR and continuous chest compressions during sustained inflations, the first dose of epinephrine was administered at 6 min [68]. 6/13 lambs achieved ROSC without epinephrine in a median (IQR) time of 210 (185–230) sec, while in the seven lambs that received epinephrine, ROSC was achieved in a median (IQR) time of 60 (45–130) sec following epinephrine administration [68]. This time to ROSC from epinephrine administration was comparable to another study in a similar model, where the median (IQR) time to ROSC following IV epinephrine was 90 (70–140) sec [38]. Collectively, results from this series of studies suggest that while not all asphyxiated cardiac arrested animal models require epinephrine to achieve ROSC, administration of epinephrine appears to hasten ROSC. 

Epinephrine administration has been shown to increase mean arterial pressure and carotid blood flow in asphyxiated bradycardic newborn lambs [66]. The hemodynamic effects of epinephrine during chest compressions in asphyxial cardiac arrest, however, do not corroborate these findings. Hemodynamic data during chest compression in perinatal asphyxiated newborn lambs did not demonstrate any significant increase in diastolic or systolic blood pressures, and no increase in carotid blood flow following epinephrine administration [38,69]. One possible explanation for the lack of effect on hemodynamics with epinephrine administration in this model may be due to the depletion of adenosine triphosphate during asphyxia, which is required to maintain vascular tone. 

In the absence of positive hemodynamic effects from epinephrine administration in the severely asphyxiated neonate, repeat epinephrine doses may potentiate adverse effects. Pharmacokinetic data has shown that repeated intravenous epinephrine administration (0.03 mg/kg) results in a cumulative increase in plasma epinephrine concentrations that can exceed 1000 ng/mL after four doses [38]. Also, very high plasma epinephrine concentrations (>700 ng/mL) were observed following ROSC in lambs that received repeated doses of ETT epinephrine. These data suggest that epinephrine administered into the lungs is not well absorbed during resuscitation. The lungs can function as a depot until pulmonary blood flow increases upon ROSC resulting in a sharp rise in epinephrine plasma concentrations. Repeated epinephrine doses were associated with a higher risk of tachyarrhythmia. Interestingly, in McNamara et al.’s study, piglets that were randomized to receive low dose epinephrine were noted to have more frequent VF on echocardiogram, and required a greater number of shocks and higher joules [56].

## 4. Epinephrine Flush Volume

**Figure 6:**
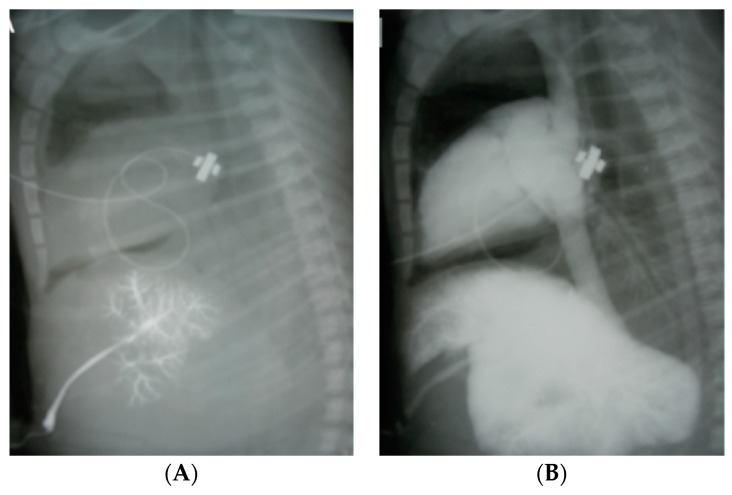
X-ray showing distribution of contrast following low- and high-volume flush through a low-lying umbilical venous catheter following 30 sec of chest compressions in term cardiac-arrested lambs. (**A**) Administration of 1 mL flush of Omnipaque shows the contrast remained in the portal venous system. (**B**) Increasing the Omipaque flush solution to 10 mL resulted in a much better distribution of contrast into the heart and great vessels. Copyright Satyan Lakshminrusimha.

The current NRP guidelines recommend a 0.5–1.0 mL normal saline flush following epinephrine administration from a low lying UVC [24]. It is unclear whether this volume is sufficient to propel epinephrine from the umbilical vein into the right atrium to reach the circulation and may deposit most of the drug in the umbilical vein and liver (Figure 6). In a perinatal asphyxiated cardiac arrest lamb model, subjects were randomized to receive a (1) low-volume 1 mL normal saline flush or (2) high-volume 10 mL (approximately 3 mL/kg) normal saline flush following administration of IV epinephrine, 0.03 mg/kg [70]. Lambs that received a high-volume flush had 100% ROSC success (3/3 lambs) following the first dose of epinephrine compared to 33% ROSC in lambs who were given a low-flush volume (1/3 lambs; *p* > 0.05). In addition, the median time (IQR) to ROSC was shorter in the high-volume flush at 40 sec (35–50 s) compared to 48 sec (42–54 s) in the low-volume flush (*p* > 0.05) [70].

## 5. Alternate Medication in Neonatal Resuscitation

Given the potential adverse effects of epinephrine and that epinephrine is the only recommended vasoactive drug to be administered during bradycardia or asystole by NRP, there is great interest in finding alternative vasoconstrictors to be used during neonatal resuscitation. Vasopressin was first proposed as a resuscitation agent after endogenous vasopressin concentrations were found to be higher in successfully resuscitated patients compared with those who died [71]. The evidence for vasopressin use in cardiac arrest, however, has been contentious. Animal experimental models in cardiac arrest have demonstrated improved survival after vasopressin administration compared to epinephrine [72,73,74,75], though clinical trials have not demonstrated improved outcomes and the use of vasopressin is currently not recommended for pediatric or adult cardiac arrest [28,41]. Unlike epinephrine, vasopressin is not a direct myocardial stimulant and does not significantly increase myocardial oxygen demand. In Mcnamara and colleagues’ study in asphyxiated newborn piglets, vasopressin was shown to improve survival, lower post-resuscitation troponin, and less hemodynamic compromise compared to epinephrine [56]. In contrast, in a perinatal asphyxiated cardiac arrest lamb model, vasopressin (0.4 U/kg IV) compared to epinephrine (0.03 mg/kg IV resulted) resulted in a lower incidence of ROSC (3/9 vs. 7/10, respectively), as well as a longer time to achieve ROSC (13 ± 6 min vs. 8 ± 2 min, respectively). Furthermore, vasopressin caused coronary vasoconstriction (37 ± 44 g/g), whereas epinephrine dilated coronary arterial rings (−16 ± 12 g/g, *p* < 0.05). A vasoconstriction response to epinephrine was higher compared to vasopressin in carotid (162 ± 64 vs. 49 ± 52 g/g, *p* = 0.02) and pulmonary arterial rings (19 ± 6 vs. 4 ± 9g/g, *p* = 0.01) [76]. Clinical studies with neurodevelopmental follow-up comparing epinephrine and vasopressin during neonatal resuscitation is warranted.

## 6. Conclusions

In an era fueled by scientific research that is growing at an exponential rate, we strive to assimilate the vast evidence available to provide the best care to our patients. In the field of neonatal resuscitation, particularly pertaining to optimizing CPR and drug delivery in the most severely asphyxiated newborns, there remain important gaps in knowledge that remain to be addressed. The infrequent need for aggressive resuscitation in newborns has prevented the execution of large randomized clinical trials. As a result, the current recommendations are extrapolated from adult, animal or manikin studies that do not adequately represent the transitioning circulation and fluid-filled lungs characteristic of newborns. A novel perinatal asphyxial cardiac arrest newborn lamb model has provided new evidence on the pharmacokinetics and hemodynamics of epinephrine during neonatal resuscitation. Future studies assessing physiologic parameters (including coronary and ductal blood flow) of variable doses and routes (IO, intramuscular) of vasoactive drug administration will provide critical insights to further advance the field of neonatal resuscitative medicine.
